# The predictive value of Naples prognostic score for patients with locally advanced non-small cell lung cancer undergoing surgery after neoadjuvant chemotherapy

**DOI:** 10.3389/fimmu.2025.1578896

**Published:** 2025-05-22

**Authors:** Yanfei Zhang, Chunyan Tang, Min Yang, Shixuan Li, Fangchao Li, Yang Wang, Li Qi, Jingjing Li

**Affiliations:** ^1^ Department of Oncology, Affiliated Hospital of Shandong Second Medical University, School of Clinical Medicine, Shandong Second Medical University, Weifang, Shandong, China; ^2^ Jinming Yu Academician Workstation of Oncology, Shandong Second Medical University, Shandong, China; ^3^ Department of Infectious Diseases, Affiliated Hospital of Shandong Second Medical University, School of Clinical Medicine, Shandong Second Medical University, Weifang, Shandong, China; ^4^ Medical Center, University of Chicago, Cancer Center, Medical Center, Chicago, IL, United States

**Keywords:** Naples prognostic score, NSCLC, neoadjuvant chemotherapy, NPS, LMR

## Abstract

**Objective:**

To evaluate the prognostic significance of the Naples Prognostic Score (NPS) in patients with locally advanced non-small cell lung cancer (NSCLC) after neoadjuvant chemotherapy and surgery.

**Methods:**

A retrospective review was done of 126 patients with locally advanced NSCLC who were surgically treated Affiliated Hospital of Weifang Medical University. from September 2012 to April 2019. According to the neutrophil-to-lymphocyte ratio (NLR), lymphocyte-to-monocyte ratio (LMR), albumin, and total cholesterol before neoadjuvant chemotherapy, NPS was divided into separate groups: group 0, group 1, and group 2. Kaplan-Meier method was used to analyze survival curves for the NPS. Univariate and multivariate Cox analysis of overall survival (OS) and progression-free survival (PFS) was then conducted.

**Results:**

This study included 60 male and 66 female patients, with the median age being 59 (59.94 ± 11.77). Based on the NPS system, the three groups were divided: Group 0, 41(32.5%) patients; Group 1, 55(43.7%) patients; and Group 2, 30(23.8%) patients. Smoking status (P=0.032) and KPS score (P=0.018) were significantly different among the three NPS groups, but it had no statistical relevance in regards to gender (P=0.849), age (P=0.474), clinical stage (P=0.101), pathology (P=0.819), tumor location (P=0.304), degree of differentiation (P=0.889), surgical method (P=0.436), chemotherapy (P=0.718), postoperative complications (P=0.177) or CEA level (P=0.447). Univariate Analysis showed that clinical stage (P=0.004), KPS score (P=0.003), surgery approach (P=0.042) and NPS (Group 2 vs. Group 0, P< 0.001; Group 1 vs. Group 0, P=0.005) were predictors of OS in patients with locally advanced NSCLC, and that clinical stage (P=0.005), KPS score (P=0.002), and NPS (Group 2 vs. Group 0, P< 0.001; Group 1 vs. group 0, P=0.001) were significantly associated with PFS. Based on the positive results of univariate analysis, we performed multivariate analysis. Multivariate Cox Regression showed that the NPS was a significant independent predictor of worse OS (Group 2 vs. Group 0, P=0.006; Group 1 vs. group 0, P=0.017) and PFS (group 2 vs. group 0, P=0.006; Group 1 vs group 0, P=0.011).

**Conclusion:**

As a clinically accessible blood indicator, NPS has vital value in predicting the prognosis of resected locally advanced NSCLC patients receiving neoadjuvant chemotherapy and surgery.

## Introduction

Lung cancer is the second most diagnosed cancer in both men and women but the most common the leading cause in cancer related deaths (1). Lung cancer brings high economic burden for governments and families (2, 3). Most patients with early-stage NSCLC fall outside of the optimal treatment window the treatment due to lack of clinical manifestations (1). Stage III lung cancer, also known as locally advanced lung cancer, is a type of disease with very strong clinical heterogeneity (4). Patients with locally advanced NSCLC have a poor prognosis, with a 5-year survival rate of only 15%-40% (5). Therefore, there is an urgent need to stratify patients with locally advanced NSCLC according to individual heterogeneity, to explore effective and reliable prognostic indicators. In current clinical practice, researchers have confirmed that inflammation-related hematological biomarkers can be used as markers for predicting patient prognosis, including C-reactive protein (CRP), mean platelet volume/platelet count ratio (MPV/PC ratio), platelet-to-lymphocyte ratio (PLR), lymphocyte-white blood cell ratio(LWR), lymphocyte to CRP ratio (LCR) and CRP to albumin ratio (CAR) etc (6–13). Additionally, inflammatory markers such as Neutrophil-to-Lymphocyte Ratio (NLR) and lymphocyte-to-monocyte ratio (LMR), and other prognostic factors that represent or reflect the nutritional or immune status of patients have also been confirmed by various studies to be key factors in predicting the survival rate of osteosarcoma (14, 15). Besides, prognostic nutritional index (PNI) like albumin, cholesterol, controlled nutritional status (CONUT) score, systemic immune inflammation index (SII) also shows prognostic value (16–20). Therefore, a multidimensional prognostic evaluation system containing multiple markers or factors together may have better predictive efficacy than a single prognostic factor.

The Naples prognostic score (NPS) is a comprehensive prognostic scoring system, calculated according to serum albumin and total cholesterol concentrations, LMR and NLR (21, 22). As an immune and nutritional evaluation method, it has been used to predict the prognosis of various solid tumors including gastrointestinal (GI) cancers, non-small-cell-lung cancer, gallbladder cancer (23–26). Moreover, it also predicts other diseases development, such as adult asthma (21), heart failure mortality (27), pulmonary arterial hypertension (28). Up to now, the significance of the NPS prognostic score in the prognostic value of surgically resected locally advanced NSCLC patients has not been widely studied.

Herein, the purpose of this study is to evaluate the correlation between the NPS prognostic score and the clinicopathological characteristics as well as the long-term prognosis of patients with locally advanced NSCLC. Furthermore, the NPS prognostic score was compared with previously developed scoring systems and the classic TNM staging system to evaluate whether the Naples prognostic score has a predictive value for the prognosis of patients with locally advanced NSCLC who underwent surgery after neoadjuvant chemotherapy.

## Data and methods

### Clinical data

A total of 126 patients with locally advanced NSCLC who were eligible for surgery at the Affiliated Hospital of Weifang Medical College between September 2012 and April 2019 were selected. Inclusion criteria: (1) aged over 18 years; (2) no anti-tumor treatment before admission; (3) patients diagnosed with NSCLC by histological pathology. (4) Karnofsky (KPS) functional status score within 80–100 points; (5) complete peripheral blood test collected 1 week prior to treatment initiation, including neutrophils, monocytes, lymphocytes, albumin, cholesterol, tumor markers, etc. (6) no other major medical morbidities, and were otherwise deemed ideal candidates for chemotherapy and surgery. (7) patients and their families agreed to chemotherapy and surgery and signed informed consent for chemotherapy and surgery. Exclusion criteria: (1) patients with other malignant tumors. (2) patients previously treated with nonsteroidal anti-inflammatory drugs or antibiotics; (3) patients with active or chronic infectious diseases or inflammatory conditions such as blood diseases, liver diseases, and immune system diseases. This retrospective study was approved by the ethics committee of affiliated hospital of Weifang Medical University (NO. wyfy-2024-ky-490).

### Data collection

The following information of all patients collected from the electronic medical record system of the Affiliated Hospital of Weifang Medical College was included: age, sex, smoking status, KPS score, pathological type, tumor location, degree of differentiation, clinical stage, surgical method, chemotherapy regimen, postoperative complications, CEA level, overall survival (OS), and progression-free survival (PFS). In addition, serum albumin and total cholesterol levels as well as lymphocyte, neutrophil, and monocyte counts were collected 1 week prior to the initiation of neoadjuvant chemotherapy.

### NPS prognostic score

The NPS prognostic score is calculated based on plasma albumin and cholesterol levels, NLR, and LMR. The neutrophil count divided by the lymphocyte count equals the NLR, and the lymphocyte count divided by the monocyte count equals the LMR. Based on previous reports, an albumin concentration <4 mg/dL is scored as 1 point, and ≥4 mg/dL is scored as 0 point. A total cholesterol level ≤180 mg/dL is scored as 1, and a total cholesterol level >180 mg/dL is scored as 0. An NLR ≥2.96 is scored as 1 point, and an NLR <2.96 is scored as 0 point. An LMR ≤4.44 is scored as 1, and an LMR >4.44 is scored as 0. The NPS prognostic score is the sum of the plasma albumin and cholesterol levels, NLR, and LMR scores (6). According to the NPS prognostic score, patients were divided into three groups: Group 0, patients with a prognostic score of 0; Group 1, patients with a prognostic score of 1 or 2; Group 2, patients with a prognostic score of 3 or 4. The systemic inflammation score (SIS) is defined as follows: 2 points for serum albumin concentration <4 mg/dL and LMR ≤ 4.44; 0 points for serum albumin concentration ≥4 mg/dL and LMR>4.44; 1 point for serum albumin concentration <4 mg/dL or LMR ≤ 4.44.

### Treatment methods

According to the Clinical Diagnosis and Treatment Guidelines for Lung Cancer of the Oncology Branch of the Chinese Medical Association (2021 edition), senior oncology experts discussed and analyzed the treatment plans for all chemotherapy patients. Specific chemotherapy plans include: pemetrexed combined with platinum, paclitaxel combined with platinum, gemcitabine combined with platinum, and pemetrexed or paclitaxel combined with platinum chemotherapy, respectively. The specific dosage of the drug needs to be determined in combination with the patient’s body tolerance and tumor condition to determine the dosage and time of chemotherapy drugs. The conclusion of chemotherapy is then followed by a two-week resting period. If there are no surgical contraindications, then a patient is recommended to undergo surgical treatment. If the patient does have surgical contraindications, then it is recommended that the resting period be extended, and surgery can be initiated if there are any indications. est time should be appropriately extended, and surgical treatment should be performed after there are indications. The specific surgical method depends on each individual patient’s tumor characteristics. The surgical methods include thoracoscopic surgery and thoracotomy. For the choice of the two surgical methods, at least 3 thoracic surgeons will make a comprehensive consideration to ensure the safety of the operation and reduce patient trauma.

### Follow-up

All patients are followed up regularly after the initiation of treatment. According to the follow-up system regulations, patients are mainly contacted through outpatient examinations or telephone calls. The follow-up interval is every 3 months for the first 3 years and every 6 months for the next 6 years. Routine physical examinations, laboratory tests, chest and abdominal CT, cranial MRI and other imaging examinations are performed. OS is defined as the time from the first treatment to death (event) or the last follow-up (review), and PFS is defined as the time from the start of treatment to disease progression (including metastasis, recurrence or death).

### Statistical analysis

IBM SPSS Statistics 20.0 (SPSS, Inc., Chicago, IL) and Graphpad (version 8.0) were used for all statistical analyses. The receiver operating characteristic (ROC) curve analysis method was used to determine the predictive accuracy of NPS and its component parameters. The Kaplan-Meier method and Log-rank test were used to compare the differences in survival between NPS groups. Univariate and multivariate Cox proportional hazards regression analysis was used to determine prognostic factors. The hazard ratio (HR) and its 95% confidence interval (95%CI) were also calculated.

## Results

### Clinical characteristics of patients

**Figure 1:**
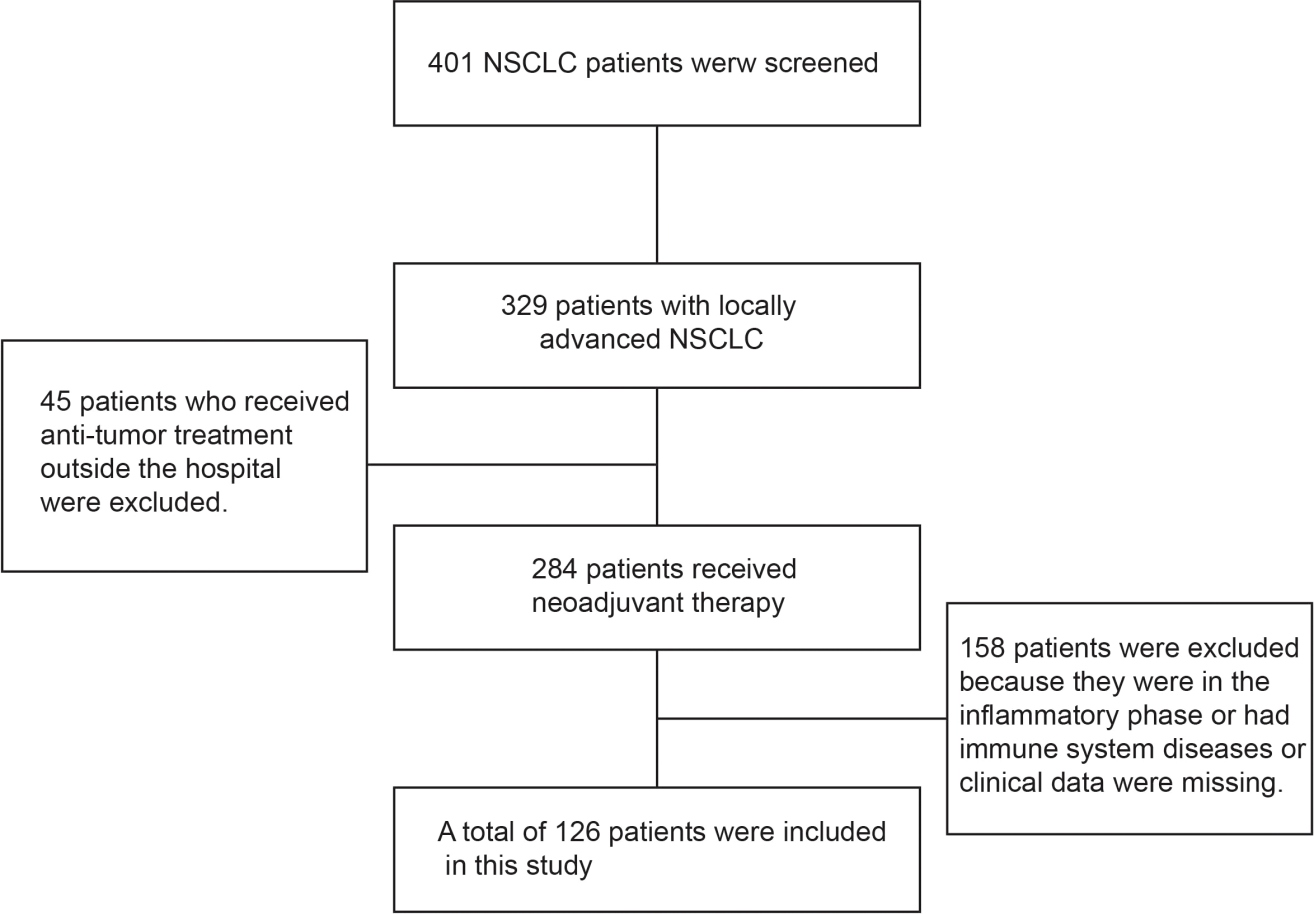
Demonstrated case screening process. Totally, 401 patients with NSCLC were screened, including 329 patients with locally advanced disease, and preliminarily excluded 45 patients who had undergone relevant anti-tumor therapy in other hospitals. A further 284 patients received neoadjuvant therapy, and 158 patients with inflammatory phase or immune system diseases or lack of clinical data were excluded, for a total of 126 patients were included in this study.

A total of 126 patients with locally advanced NSCLC were included in this study. All patients underwent surgical treatment after neoadjuvant chemotherapy, and no data were missing during follow-up (**Figure 1**). The average age of these patients at the time of treatment was 59 years (59.94 ± 11.77 years). 98 (77.8%) patients had a KPS score of 100 points, and 28 (22.2%) had a KPS score of 80–90 points. 66 (46.8%) patients had a history of smoking. There were 54 (42.9%) patients with squamous cell carcinoma and 72 (57.1%) patients with adenocarcinoma; according to the TNM staging system, there were 82 (65.1%) patients in stage IIIA and 44 (34.9%) patients in stage IIIB. All patients received neoadjuvant chemotherapy, including 59 (46.8%) patients who received paclitaxel combined with platinum, 30 (23.8%) patients who received pemetrexed combined with platinum, and 37 (29.4%) patients who received gemcitabine combined with platinum. The baseline characteristics and results of the patients are summarized in **Table 1**.

**Table 1:** Relationship between NPS prognostic score and clinicopathological characteristics in patients with locally advanced NSCLC.

Clinical characteristics	NPS		P	
	Number of cases	Group0	Group1	Group2
Gender				0.849
Female	66 (52.4%)	20 (48.8%)	30 (54.5%)	16 (53.3%)
Male	60 (47.3%)	21 (51.2%)	25 (45.5%)	14 (46.7%)
Age (years)				0.474
<60	67 (55.2%)	25 (48.6%)	27 (55.6%)	15 (63.6%)
≥60	59 (44.8%)	16 (51.4%)	28 (44.4%)	15 (36.4%)
Smoking				0.032
No	60 (53.2%)	26 (63.4%)	24 (43.6%)	10 (33.3%)
Yes	66 (46.8%)	15 (36.6%)	31 (56.4%)	20 (66.7%)
Clinical stage				0.101
IIIA	82 (65.1%)	32 (78.0%)	33 (60.0%)	17 (56.7%)
IIIB	44 (34.9%)	9 (22.0%)	22 (40.0%)	13 (43.3%)
KPS score				0.018
100	98 (77.8%)	36 (87.8%)	44 (80.0%)	18 (60.0%)
80-90	28 (22.2%)	5 (12.2%)	11 (20.0%)	12 (40%)
Pathological type				0.819
Adenocarcinoma	72 (57.1%)	25 (61.0%)	30 (54.5%)	17 (56.7%)
Squamous cell carcinoma	54 (42.9%)	16 (39.0%)	25 (45.5%)	13 (43.3%)
Tumor location				0.304
Left lung	96 (76.2%)	30 (73.2%)	40 (72.7%)	26 (86.7%)
Right lung	30 (23.8%)	11 (26.8%)	15 (27.3%)	4 (13.3%)
Degree of differentiation				0.889
High / Medium	54 (42.9%)	17 (38.6%)	23 (41.8%)	14 (46.7%)
Low	72 (57.1%)	24 (61.4%)	32 (58.2%)	16 (53.3%)
Surgical method				0.436
Open chest	56 (44.4%)	16 (39.0%)	28 (50.9%)	12 (40.0%)
VAST	70 (55.6%)	25 (61.0%)	27 (49.1%)	18 (60.0%)
Chemotherapy regimen				0.718
Paclitaxel combined with platinum	59 (46.8%)	22 (53.7%)	23 (41.8%)	14 (46.7%)
Pemetrexed combined with platinum	30 (23.8%)	10 (24.4%)	14 (25.5%)	6 (20.0%)
Gemcitabine combined with platinum	37 (29.4%)	9 (21.9%)	18 (32.7%)	10 (33.3%)
Postoperative complications				0.177
Yes	62 (49.2%)	17 (41.5%)	26 (47.3%)	19 (63.3%)
No	64 (50.8%)	24 (58.5%)	29 (52.7%)	11 (36.7%)
CEA level				0.447
Normal	66 (52.4%)	24 (58.5%)	29 (52.7%)	13 (43.3%)
Abnormal	60 (47.6%)	17 (41.5%)	26 (47.3%)	17 (56.7%)

### Relationship between NPS prognostic score and clinicopathological characteristics

According to the calculation of NPS prognostic scoring standards, 41 (32.5%) patients with a score of 0 points were included in the NPS group 0, 55 (43.7%) patients with a score of 1–2 points were included in the NPS group 1, and the remaining 30 (23.8%) patients with scores of 3 to 4 were included in NPS group 2. The NPS prognostic score is significantly correlated with some clinicopathological features. The results revealed statistically significant differences in smoking status (P=0.032) and KPS score (P=0.018) in the three groups. However, gender (P=0.849), age (P=0.474), and clinical stage (P= 0.101), pathological type (P=0.819), tumor location (P=0.304), degree of differentiation (P=0.889), surgical method (P=0.436), chemotherapy regimen (P=0.718), postoperative complications (P= 0.177) and CEA level (P=0.447) had no statistical significance among the three groups of NPS prognostic score.

### Laboratory indicator analysis

The median values of neutrophil count, lymphocyte count, monocyte count, NLR, LMR, albumin and total cholesterol were 4.58 (95%CI: 4.27-4.79) and 1.99 (95%CI: 1.83-2.18) respectively.), 0.48 (95%CI: 0.46-0.57), 2.31 (95%CI: 2.09-2.43), 4.74 (95%CI: 3.65-4.85), 4.33 (95%CI: 4.24-4.41) mg/dL and 195.00 (95% CI: 186.01-226.00) mg/dL (**Table 2**).

**Table 2:** Blood indexes.

Variables	Median (95%CI)
Serum albumin (mg/dL)	4.33 (4.24-4.41)
Total cholesterol (mg/dL)	195.00 (186.01-226.00)
Neutrophil count (10^9^/L)	4.58 (4.27-4.79)
Lymphocyte count (10^9^/L)	1.99 (1.83-2.18)
Monocyte count (10^9^/L)	0.48 (0.46-0.57)
NLR	2.31 (2.09-2.43)
LMR	4.74 (3.65-4.85)

### Survival difference analysis

**Figure 2:**
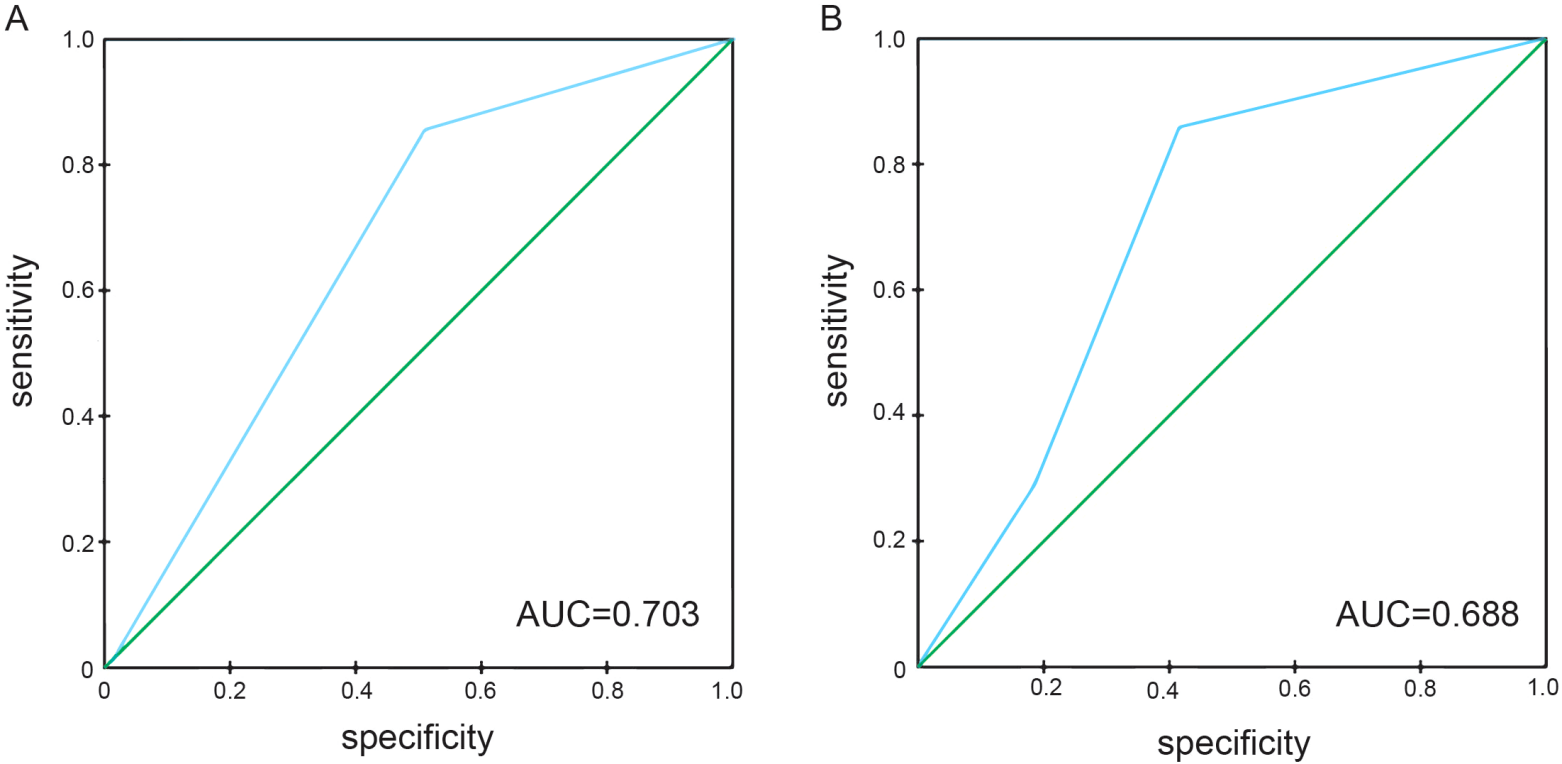
ROC curves of NPS for predicting survival prognosis in patients with locally advanced NSCLC. **(A)** The ROC curve of NPS score predicting OS in patients with locally advanced NSCLC, area under the curve AUC=0.703. **(B)** The ROC curve of NPS score predicted PFS in patients with locally advanced NSCLC, and the area under the curve AUC=0.688.

**Figure 3:**
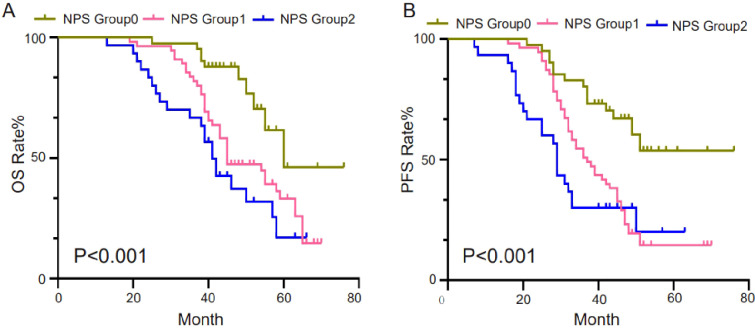
Survival curves of survival prognosis of patients involved in different groups. **(A)** For all enrolled patients, patients in the NPS 0 group had better OS compared to patients in the NPS 2, NPS 1 groups (2 groups vs 0 groups, P<0.001, 1 group vs 0 groups, P=0.005). **(B)** In terms of PFS, NPS 0 had better PFS than NPS 2 and NPS 1 (2 vs 0, P<0.001, 1 vs 0, P=0.001).

The predictive performance of NPS, NLR, LMR, albumin, and total cholesterol was evaluated by ROC curve analysis. **Table 3** shows the area under the curve (AUC), 95% confidence interval (95%CI), and P value of NPS, NLR, LMR, albumin, and total cholesterol. The results showed that the NPS prognostic score had a higher predictive value for OS (AUC=0.703) and PFS (AUC=0.688). NLR (P=0.534, P=0.527), LMR (P=0.611, P=0.555), albumin (P=0.566, P=0.562), and total cholesterol (P=0.625, P=0.654) had lower predictive values for OS and PFS. The ROC curve can clearly show the predictive ability of the NPS prognostic score (**Figure 2**). The OS and PFS survival curves of NPS prognostic scores (**Figure 3**). For all enrolled patients, patients in NPS 0 group had better OS compared with patients in NPS 2 and NPS 1 groups (group 2 vs group 0, P < 0.001, group 1 vs group 0, P = 0.005); in terms of PFS, NPS 0 group had better PFS than NPS 2 and NPS 1 groups (group 2 vs group 0, P < 0.001, group 1 vs group 0, P = 0.001).

**Table 3:** ROC curve analysis of blood indicators on OS and PFS.

	OS			PFS		
Indicators	AUC	95%CI	P	AUC	95%CI	P
NPS	0.703	0.609-0.798	<0.001	0.688	0.582-0.794	0.001
NLR	0.534	0.433-0.635	0.514	0.527	0.422-0.632	0.620
LMR	0.611	0.512-0.710	0.033	0.555	0.449-0.660	0.413
Albumin	0.566	0.466-0.666	0.204	0.562	0.459-0.665	0.252
Total cholesterol	0.625	0.527-0.723	0.016	0.654	0.556-0.752	0.005

### Single factor and multi-factor analysis

To explore the relationship between various clinicopathological factors and OS and PFS in patients with locally advanced NSCLC, univariate analysis was performed for each factor (**Table 4**). Univariate analysis showed clinical stage (P=0.004), KPS score (P=0.003), surgical method (P=0.042) and NPS prognostic score (group 2 vs group 0, P<0.001; group 1 vs group 0, P= There is a significant correlation between 0.005) and OS. However, gender (P=0.506), age (P=0.460), smoking status (P=0.744), pathological type (P=0.969), tumor location (P=0.419), and degree of differentiation (P=0.323) were not found and CEA level (P=0.806) were related to OS. Clinical stage (P=0.005), KPS score (P=0.002) and NPS prognostic score (group 2 vs group 0, P<0.001; group 1 vs group 0, P=0.001) were related to PFS. Indicators that were meaningful in the univariate analysis of OS (NPS prognostic score, clinical stage, KPS score, and surgical approach) were included in the multivariate analysis. Multivariate analysis results revealed that NPS prognostic score was an independent prognostic factor for OS (group 2 vs group 0, P=0.006; group 1 vs group 0, P=0.017); however, clinical stage (P=0.084), KPS score (P=0.057) and surgical method (P=0.141) were not statistically significant. Based on the results of PFS single-factor analysis, clinical stage, KPS score (P=0.057) and NPS prognostic score were included in multi-factor analysis, and the results showed that NPS prognostic score (group 2 vs group 0, P=0.006; group 1 vs 0 group, P=0.011) is an independent prognostic predictor of PFS in patients with locally advanced NSCLC (**Table 5**).

**Table 4:** Univariate analysis of OS and PFS in patients with locally advanced NSCLC.

Clinical characteristics	OS		PFS	
	HR (95% CI)	P	HR (95% CI)	P
Gender				
Female	1.000		1.000	
Male	0.852 (0.531-1.367)	0.506	0.987 (0.641-1.522)	0.954
Age (years)				
<60	1.000		1.000	
≥60	1.194 (0.746-1.911)	0.460	1.427 (0.926-2.201)	0.107
Smoking				
No	1.000		1.000	
Yes	0.925 (0.578-1.479)	0.744	0.887 (0.576-1.367)	0.588
Clinical stage				
IIIA	1.000		1.000	
IIIB	1.984 (1.240-3.174)	0.004	1.888 (1.217-2.929)	0.005
KPS score				
100	1.000		1.000	
80-90	2.112 (1.281-3.482)	0.003	2.098 (1.308-3.365)	0.002
Pathological type				
Adenocarcinoma	1.000		1.000	
Squamous cell carcinoma	1.009 (0.628-1.623)	0.969	1.212 (1.785-1.869)	0.386
Tumor location				
Left lung	1.000		1.000	
Right lung	0.794 (0.453-1.390)	0.419	1.561 (0.985-2.472)	0.058
Degree of differentiation				
High / Medium	1.000		1.000	
Low	1.273 (0.789-2.054)	0.323	0.949 (0.616-1.463)	0.813
Surgical method				
Open chest	1.000		1.000	
VAST	0.614 (0.383-0.983)	0.042	0.676 (0.439-1.041)	0.076
Chemotherapy regimen				
Paclitaxel combined with platinum	1.000		1.000	
Pemetrexed combined with platinum	0.932 (0.500-1.738)	0.825	0.929 (0.530-1.627)	0.796
Gemcitabine combined with platinum	1.007 (0.586-1.732)	0.979	1.312 (0.800-2.152)	0.283
Postoperative complications				
Yes	1.000		1.000	
No	0.739 (0.461-1.184)	0.209	0.842 (0.547-1.295)	0.434
CEA level				
Normal	1.000		1.000	
Abnormal	1.061 (0.663-1.697)	0.806	1.004 (0.652-1.547)	0.985
NPS prognostic score				
Group 0	1.000		1.000	
Group 1	2.750 (1.368-5.532)	0.005	2.789 (1.556-5.000)	0.001
Group 2	4.025 (1.893-8.557)	<0.001	3.764 (1.946-7.281)	<0.001

**Table 5:** Multivariate analysis of OS and PFS in patients with locally advanced NSCLC.

Clinical characteristics	OS		PFS	
	HR (95% CI)	P	HR (95% CI)	P
Clinical stage				
IIIA	1.000		1.000	
IIIB	1.534(0.944-2.495)	0.084	1.638(1.016-2.640)	0.043
KPS score				
100	1.000		1.000	
80-90	1.666(0.984-2.821)	0.057	1.667(0.977-2.839)	0.061
Surgical method				
Open chest	1.000			
VAST	0.696(0.429-1.128)	0.141		
NPS prognostic score				
Group 0	1.000		1.000	
Group 1	2.364(1.163-4.804)	0.017	2.483(1.229-5.017)	0.011
Group 2	3.019(1.377-6.620)	0.006	2.998(1.364-6.587)	0.006

## Discussion

Inflammation related factors play important roles in the occurrence and development of tumors (29). Systemic inflammation is an essential component of the tumor microenvironment (30–32). For instance, acute inflammatory response products promote tumor cell proliferation, metastasis and invasion through various pathways. Inflammatory mediators like Interleukin-6 (IL-6) and Tumor Necrosis Factor-alpha (TNF-alpha) activate STAT3 and NF-κB signaling pathways to promote cancer cell proliferation (33, 34). Besides, inflammatory signals can induce epithelial-to-mesenchymal transition (EMT) (35), angiogenesis (36) and extracellular matrix (ECM) remodeling (37) for cancer cell metastasis and invasion. Besides, inflammatory factors also promote tumor angiogenesis by upregulating the expression of vascular endothelial growth factor (38–42). In current clinical practice, researchers have confirmed that inflammation-related hematological biomarkers can be used as markers for predicting patient prognosis, including C-reactive protein (CRP), NLR, LMR, mean platelet volume to platelet ratio (MPV/PCT), etc (6–8). In addition to inflammatory markers such as NLR and LMR, other prognostic factors that represent or reflect the nutritional or immune status of patients have also been confirmed by various studies to be key factors in predicting the survival rate of other malignancies, such as osteosarcoma, including prognostic nutritional index (PNI), albumin, cholesterol, controlled nutritional status (CONUT) score, systemic immune inflammation index (SII), etc (16–20). Therefore, multi-dimensional prognostic evaluation systems may have better predictive power than a single prognostic factor. Recently, the NPS prognostic score, which is calculated from serum albumin, total cholesterol concentration, LMR, and NLR, has attracted attention. The NPS prognostic score is a comprehensive prognostic scoring system that includes the currently used inflammatory and nutritional prognostic markers. However, the significance of the NPS prognostic score in the prognostic value of locally advanced NSCLC patients who underwent surgical resection has not been extensively studied. During the preparation of the manuscript for this study, Zou et al. also reported the value of the NPS prognostic score in the survival prognosis of patients with locally advanced non-small cell lung cancer who underwent resection after neoadjuvant therapy (43).

In this research, we evaluated the correlation between the NPS prognostic score and the clinicopathological characteristics as well as long-term prognosis of patients with locally advanced NSCLC. In addition, the NPS prognostic score was compared with previously developed scoring systems and the classic TNM staging system to evaluate its performance. Our results are consistent with previous studies. Our study revealed that the NPS prognostic score combines inflammation, nutrition and immune parameters, and is better than a single indicator in predicting the survival of patients with locally advanced NSCLC who received surgery after neoadjuvant chemotherapy, comparing to other prognostic indicators related to immunity and nutrition.

Although our study has achieved certain results, this study still has the inevitable limitation of sample size. To more clearly predict the prognosis of lung cancer or even other types of cancer, a larger sample size is needed in the future. First, this study is a retrospective study with a small sample from a single center. Secondly, among the components of NPS, including NLR, LMR, albumin and total cholesterol, the cutoff values of the above indicators are determined by previous literature, and this article does not set its own cutoff values based on our study. Secondly, all patients with locally advanced NSCLC included in this study received neoadjuvant chemotherapy and surgical treatment, but some patients received chemoradiotherapy and some patients received chemotherapy alone after surgery. There are differences in treatment methods, which may affect the prediction of survival by NPS to a certain extent. The patients selected for this study had strict inclusion criteria, but laboratory indicators such as serum albumin, total cholesterol, neutrophils, lymphocytes, and monocytes are easily affected by various factors and cannot be accurately controlled.

In summary, the NPS prognostic score is a simple and reliable preoperative prognostic scoring system that can independently predict the OS and PFS of patients with locally advanced NSCLC who undergo surgical treatment. The NPS prognostic score reflects the importance of biomarkers related to inflammation and nutrition. By reflecting the inflammatory and nutritional status of tumor patients, it can accurately stratify patients into risk groups, play a positive role in adjusting treatment strategies, and enable patients to obtain survival benefits.

## Data Availability

The original contributions presented in the study are included in the article/supplementary material. Further inquiries can be directed to the corresponding author.
